# Proteases as Insecticidal Agents

**DOI:** 10.3390/toxins2050935

**Published:** 2010-05-05

**Authors:** Robert L. Harrison, Bryony C. Bonning

**Affiliations:** 1 Invasive Insect Biocontrol and Behavior Laboratory, USDA Agricultural Research Service, Plant Sciences Institute, 10300 Baltimore Avenue, Beltsville, Maryland 20705, USA; Email: Robert.L.Harrison@ars.usda.gov; 2 Department of Entomology, Iowa State University, 418 Science II, Ames, IA 50011-3222, USA

**Keywords:** insecticides, basement membrane, cuticle, peritrophic matrix, plant defense, microbial defense

## Abstract

Proteases from a variety of sources (viruses, bacteria, fungi, plants, and insects) have toxicity towards insects. Some of these insecticidal proteases evolved as venom components, herbivore resistance factors, or microbial pathogenicity factors, while other proteases play roles in insect development or digestion, but exert an insecticidal effect when over-expressed from genetically engineered plants or microbial pathogens. Many of these proteases are cysteine proteases, although insect-toxic metalloproteases and serine proteases have also been examined. The sites of protease toxic activity range from the insect midgut to the hemocoel (body cavity) to the cuticle. This review discusses these insecticidal proteases along with their evaluation and use as potential pesticides.

## 1. Toxic Proteins as Insecticidal Agents

The use of classical chemical insecticides to control agricultural pests poses hazards to human health, other non-target species including beneficial insects such as pollinators, and the environment. Indiscriminate use of chemical insecticides can also select for pest populations with insecticide resistance. Even the newer insecticidal compounds with less-threatening toxicological profiles can have adverse ecological and environmental impacts [1]. The development of insecticide resistance and the growing awareness of ecological and environmental problems caused by classical chemical insecticides have spurred research into biologically-based, environmentally benign alternatives. Among the compounds identified during the course of this research are proteins from microbes, predators, and plants that have toxicity that is specific to insects. Most prominent among these insecticidal proteins are the *Bt* crystal (Cry) δ-endotoxin proteins produced by *Bacillus thuringiensis* that form crystalline inclusions during sporulation [2]. Cry proteins bind to receptors and insert into the membranes of insect midgut epithelial cells, forming pores and causing cellular lysis and fatal damage to the midgut epithelium [3]. Application of *Bt* toxins towards insect pest management has involved both spray formulations of *B. thuringiensis* crystals/spores and crops genetically engineered to express *Bt* toxin genes. The widespread planting of *Bt* toxin-expressing maize and cotton has significantly reduced pesticide application, with the associated decrease in environmental impact [4]. A number of other insecticidal proteins have been identified which also target the insect midgut, including additional lytic pore-forming proteins from *B. thuringiensis* such as vegetative insecticidal proteins (vip) and cytolytic proteins (cyt) [3,5,6], cholesterol oxidase [7], biotin-binding proteins [8], toxin complexes produced by nematode symbiotic bacteria from the genus *Photorhabdus* [9], ribosome-inactivating proteins from plants [10], protease inhibitors [11], and chitinases [12].

There has also been a significant amount of research on insecticidal proteins with targets lying beyond the gut. The most studied among these are insect-selective toxins found in the venoms of predatory invertebrates, particularly scorpions, but also mites, spiders, and sea anemones [13,14,15]. 

These peptides are neurotoxins that act on ion channels on axonal membranes, disrupting impulse transmission and causing paralysis. Effective delivery of these toxins to their targets has been achieved by genetic engineering of insect viruses (baculoviruses) [16] or entomopathogenic fungi [17] to express the toxins, and by fusion of a toxin with an appropriate delivery system [18,19]. Baculovirus infection of the tracheal system servicing the central nervous system of caterpillars ensures that a supply of synthesized, secreted toxin is available in close proximity to axonal membranes [20]. Other proteins that serve as hormones also exert a toxic effect on insects when expressed from a recombinant baculovirus [16].

Proteases are logical candidates for use as insecticidal agents. One would expect that with insects, as with other animals, proteolytic enzymatic activity can target and destroy essential proteins and tissues to an extent that mortality would result. Indeed, proteases have evolved in plants to defend against herbivorous insects. In microbial pathogens of insects, proteases often play a role in pathogenicity towards the host insect. Proteases have been found as components of the venoms of arthropod predators of insects. Because of what proteases do, even proteases that have not evolved to play a role as a toxin can still have an insecticidal effect when present at the wrong time, the wrong place, and/or the wrong quantity within an insect. This situation has been observed with microbial pathogens that have been engineered to over-express an otherwise innocuous protease during infection and growth of the pathogen within an insect. Hence, this review discusses not only proteases that have evolved as toxins of insects in different contexts, but also proteases that do not normally act as toxins of insects, but have a toxic effect when expressed by a vector (Table 1). A separate review in this volume by Rodou and colleagues provides an in-depth overview of the insecticidal proteases of the bacterium *Photorhabdus luminescens*. Related to the use of proteolytic enzymes as insecticidal agents is exploitation of the insecticidal impact of the lack of proteases, such as plant expression of inhibitors of herbivore gut proteases [21], and the use of insect-derived, trypsin-modulating oostatic factor that inhibits trypsin biosynthesis in the insect gut [22,23]. The insecticidal effects of protease inhibition will not be considered in the current review. 

**Table 1:** Proteases with insecticidal activity.

Target tissue	Class of protease		
	*Cysteine protease*	*Metalloprotease*	*Serine protease*
*Midgut* (including peritrophic matrix)	*Zea mays* Mir1-CP	Baculovirus enhancins	Trypsin
	*Carica papaya* and *Ficus virgata* latex proteases	Bacterial (*B. thuringiensis*) enhancins	
	Baculovirus V-CATH	*B. thuringiensis* InhA2	
*Cuticle*			*Metarhizium anisopliae* PR1A (subtilisin-like)
			*Beauveria bassiana* CDEP1
*Hemocoel* (including basement membrane and the prophenoloxidase cascade)	*Sarcophaga peregrina* ScathL (cathepsin L-like)	*Eulophus pennicornis* reprolysin	
	*Delia coarctata* DcCathL	*B. thuringiensis* InhA	
	*Helicoverpa armigera* cathepsin B-like		
	*Tuberaphis styraci* cathepsin B		

## 2. Proteases with Toxicity towards Insects

### 2.1. Proteases that target the peritrophic matrix

**Figure 1:**
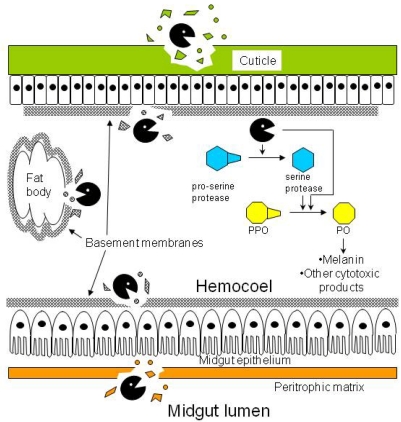
Diagram of generic insect anatomy, showing targets sites of insecticidal proteases (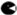). Besides degrading the cuticle, basement membranes, and peritrophic matrix, proteases in the hemocoel may convert prophenoloxidase (PPO) to phenoloxidase (PO) either directly or indirectly by activating the cascade of serine proteases that lead to the conversion step.

The peritrophic matrix (PM) is a mesh of chitin fibrils linked to glycoproteins and proteoglycans that lines the midgut epithelium of most insects [24] (Figure 1). The PM is analogous to the protective mucosal layer that lines the digestive tracts of mammals. The PM acts as a mechanical barrier that protects the midgut epithelium from abrasion by the insect diet and blocks access of ingested insect pathogens and toxins to the midgut epithelium. The PM also separates and organizes digestive processes within the midgut. Chemical compounds that disrupt the PM or block its formation or regeneration can lead to a retardation of insect larval growth and even mortality due to the inability of the exposed, damaged midgut epithelium to take up nutrients [25,26]. Thus, the insect PM is a desirable target for insecticidal proteins [27].

Some baculoviruses carry genes that encode zinc metalloproteases known as enhancins [28]. These proteases, previously known as “synergistic factors” [29], promote baculovirus infection of lepidopteran larvae by digesting PM proteins, making the PM more permeable to baculovirus virions [30,31,32]. Enhancins specifically degrade a PM protein known as invertebrate intestinal mucin (IIM) [33]. In addition to promoting viral infection of the midgut epithelium, enhancins can be toxic to insects when expressed outside of the context of a baculovirus infection. The expression of enhancins in transgenic plants results in a retardation in growth and mortality of herbivorous lepidopteran larvae that feed on the plants [34,35]. Enhancin in this context also has been reported to augment the insecticidal effect of *Bt* Cry toxins, presumably by increasing exposure of the midgut epithelium to the crystal toxins [36].

Homologs of baculovirus enhancin genes have been identified in the genomes of the bacteria *Yersinia pestis*, *Bacillus anthracis*, *Bacillus thuringiensis*, and *Bacillus cereus* [37,38]. Only one of the *Bacillus* spp. enhancin-like genes, the *bel* gene from *B. thuringiensis*, was found to be required for normal levels of Cry protein toxicity against lepidopteran larvae [39]. Purfied *bel*-encoded protease enhanced the oral toxicity of Cry toxin and was found to digest IIM proteins of *T. ni* and *Helicoverpa armigera* larvae [39]. The acrystalliferous *B. thuringiensis* strain 407 (Cry^_^) encodes a metalloprotease, InhA2, which is unrelated to Bel. InhA2 was required for the synergistic enhancement of oral toxicity of CryIC crystals observed when waxmoth (*Galleria mellonella*) larvae were co-inoculated with crystals and *B. thuringiensis* strain 407 spores [40,41]. It is unknown if InhA2 degrades PM proteins or a different set of proteins such as those in the extracellular matrix. 

A plant-encoded enzyme has also been found that targets the PM of insect pests. Inbred maize lines resistant to lepidopteran larval herbivory were found to produce a 33 kDa papain-like cysteine protease, Mir1-CP, in response to feeding by larvae of the fall armyworm, *Spodoptera frugiperda* [42,43,44]. Larvae feeding on calluses transformed with a Mir1-CP expression construct exhibited growth inhibition as seen for larvae fed on the resistant maize lines [45]. Scanning electron microscopy of the midguts of larvae fed on maize plants or calluses expressing Mir1-CP revealed cracks and perforations in the PM [46]. PM dissected from *S. frugiperda* and other insect species could also be degraded by recombinant Mir1-CP purified from baculovirus vector-infected *S. frugiperda* larvae [47]. Purified recombinant Mir1-CP kills lepidopteran larvae and can enhance the toxicity of *Bt* Cry toxins [48], similar to baculovirus-expressed enhancin [36] and bacterial homologs [39]. 

Similarly, cysteine proteases in the latex of papaya (*Carica papaya*) and a wild fig (*Ficus virgata*) were found to kill or retard the growth of larvae from three different lepidopteran species [49]. In this case, Konno and co-workers suggested that the toxic effect of the proteases was due to the very high concentration of the proteases in the latex of these plants. Other plant cysteine proteases have been identified by microarray and proteomics studies of tobacco (*Nicotiana attenuata*) and *Arabidopsis thaliana* as having a potential role in defense against lepidopteran herbivores [50].

Baculoviruses encode a cathepsin L-like protease, V-CATH, which is required for late-infection and postmortem liquefaction of the internal anatomy and weakening of the cuticle of baculovirus-infected lepidopteran larvae [51,52]. Unlike enhancin, V-CATH does not normally target the peritrophic matrix during baculovirus infection. However, expression of V-CATH in transgenic tobacco plants was found to retard the growth of *Helicoverpa armigera* (Old World bollworm) larvae that fed on the plants, suggesting that this cysteine protease may also damage or disrupt the PM. While enhancin metalloproteases and a variety of cysteine proteases can degrade PM proteins, serine proteases do not appear to be capable of degrading PM proteins in *in vitro* assays [53,54]. In a study testing the oral toxicity of five different proteases to pea aphids (*Acyrthosiphon pisum*) when included as part of an artificial diet, trypsin was the second most toxic protease, with an IC_50_ of 22 μg/mL [55]. As aphids lack peritrophic matrices, the mode of insecticidal action for the ingested trypsin is unclear, but may represent non-specific degradation of gut epithelial proteins. 

### 2.2. Proteases that target the cuticle

The cuticle is the extracellular layer of the integument, the outer covering of insects and other arthropods, and is secreted by the insect epidermis. The cuticle covers the whole of the outside of the insect as well as the foregut, hindgut, and tracheal invaginations. The cuticle occurs in layers, typically with a waxy epicuticle covering an exocuticle and endocuticle consisting of protein, lipid, and chitin cross-linked to a varying degree to provide elasticity and hardness [56].

The entomopathogenic fungi *Metarhizium anisopliae* and *Beauveria bassiana* infect a wide range of insects and related arthropods and are used in many insecticidal biocontrol products [57]. Fungal infection is initiated when spores or conidia come into contact with the cuticle of a suitable host. Under favorable environmental conditions, germination occurs and specialized structures for penetrating the cuticle develop. These events are accompanied by the synthesis and secretion of a host of cuticle-degrading enzymes, particularly chitinases and proteases [58,59,60]. Several fungal protease genes that are up-regulated in response to cuticle exposure encode proteinase K-like class II subtilisins [61]. Of these, the *M. anisopliae* protease PR1A digests cuticle proteins and is essential for virulence and cuticle penetration [62,63,64]. Expression of this enzyme is repressed in the presence of readily catabolizable carbon and nitrogen sources, but induced in the presence of insect cuticle [64,65,66]. 

A clone of *M. anisopliae* which was genetically modified with additional copies of the *pr1a* gene under the control of a constitutive promoter killed larvae of the tobacco hornworm, *Manduca sexta* 25% faster than wild-type *M. anisopliae* [67]. While expression of PR1A is normally turned off when the invading fungus enters the hemocoel [68], the expression of PR1A in the hemocoel of larvae infected with the recombinant fungus resulted in degradation of hemolymph proteins and melanization of the hemolymph and the internal anatomy of the larvae. Injection of purified PR1A into the hemocoel of *M. sexta* larvae also killed larvae and caused melanization. Hence, although the normal mode of action of PR1A is to degrade cuticle proteins and facilitate the cuticular penetration of invading fungi, PR1A is also toxic to insects when introduced into the hemocoel.

Melanin formation in insects is a normal process that takes place during post-molt hardening and darkening of the insect cuticle and in response to wounding or the appearance of foreign matter in the insect [69]. The enzyme phenoloxidase catalyzes key steps in a series of reactions leading to the production of melanin from tyrosine. Phenoloxidase is synthesized as an inactive zymogen prophenoloxidase, which is activated in response to developmental and environmental cues by a cascade of trypsin-like serine proteases [70]. The PR1A protease likely triggered melanization of larvae by activating the proteolytic cascade that leads to the cleavage and activation of prophenoloxidase. The activation of prophenoloxidase is under tight control due to the toxic nature of the products and by-products of phenoloxidase, which include quinones and oxygen free radicals [71,72]. Unregulated phenoloxidase activity and/or non-specific proteolysis of proteins within the hemocoel by PR1A may have contributed to its toxicity.

Recombinant *B. bassiana* clones engineered to constitutively express PR1A exhibited similar toxicity when used to infect larvae of the pine caterpillar, *Dendrolimus punctatus*, and the wax moth, *G. mellonella* [73]. Larvae infected with these clones exhibited extensive melanization. These clones had also been engineered to express the scorpion peptide neurotoxin AaIT, but whereas *B. bassiana* clones that expressed only AaIT caused contractile paralysis in larvae that were infected with them, the fungal clones expressing both AaIT and PR1A did not cause paralysis. Medium from cultures of the AaIT-expressing fungus caused paralysis when injected into larvae, but the paralytic activity vanished if the medium was mixed with PR1A-containing medium, suggesting that PR1A was degrading AaIT. *B. bassiana* engineered to constitutively express a *B. bassiana* PR1A-like protease (CDEP1) killed *G. mellonella* larvae 12.5% faster than wild-type *B. bassiana* [74]. Additional clones in the same study were produced which constitutively expressed a fusion protein consisting of CDEP1 linked to the N-terminus of a *B. bassiana* chitinase, CHIT1. This clone killed larvae 67% faster than wild-type and also faster than clones expressing only CHIT1, indicating that a synergistic reduction in survival time was obtained by expressing a fusion protein with both proteolytic and chitinolytic activities. However, melanization was not reported in larvae infected with either the CDEP1-expressing recombinant fungus or the fungus expressing the CDEP1:CHIT1 fusion.

### 2.3. Proteases that target the basement membrane

Basement membranes (BMs), also referred to as basal laminae, are extracellular sheets of proteins that surround the tissues of all animals, providing structural support, a filtration function, and a surface for cell attachment, migration, and differentiation [75]. The BM has been reported to act as a physical barrier to the movement of viruses [76,77,78,79,80]. Given the size exclusion limit of insect BMs [81] and the size of baculovirus virions [82], it seems likely that the BM would also act as a physical barrier to baculovirus infection and dissemination, and the results of some studies support this idea [83,84,85]. 

The time taken for baculoviruses to kill insect pests has been cited repeatedly as a disadvantage hindering their use and commercialization as pest management tools [16]. To overcome this disadvantage, baculoviruses have been engineered with insect-selective toxins or development-disrupting enzymes and hormones [86]. Expression of these genes causes a cessation of feeding and the death of the infected host faster than the time taken for the infected host to succumb to wild type baculovirus infection. Given their size exclusion properties, BMs are a logical target for enzymes that enhance baculovirus insecticidal efficacy [87]. 

To evaluate the potential for BM-degrading enzymes to enhance the insecticidal efficacy of baculoviruses, the nucleopolyhedrovirus *Autographa californica* multiple nucleopolyhedrovirus (AcMNPV) was engineered to express three different BM protein-degrading proteases, including two vertebrate matrix metalloproteases (rat stromelysin, human gelatinase A) and a cathepsin L, ScathL, from the flesh fly, *Sarcophaga peregrina* [88]. Expression of the ScathL protease had the most profound effect on baculovirus insecticidal activity, with the median survival time of infected larvae of the tobacco budworm, *Heliothis virescens* reduced to approximately 50% that of wild-type virus-infected larvae. Viruses expressing this enzyme killed larvae faster than viruses expressing scorpion peptide neurotoxins from the same promoter. ScathL had been reported to specifically digest large (>200 kDa) proteins in the BMs of imaginal discs and larval brain of flesh flies during larval development [89,90,91], but the high level of expression of ScathL from a strong, late-phase viral promoter (*p6.9*) augmented the virulence of an infecting baculovirus and greatly reduced survival time of infected larvae. In contrast, expression from a weaker, early-phase viral promoter (*ie1*) had no impact on survival time of infected hosts, suggesting that the expression level of the protease was an important determinant of the insecticidal effect. The viruses expressing vertebrate matrix metalloproteases showed no improvement in virulence relative to the wild type virus. 

**Figure 2:**
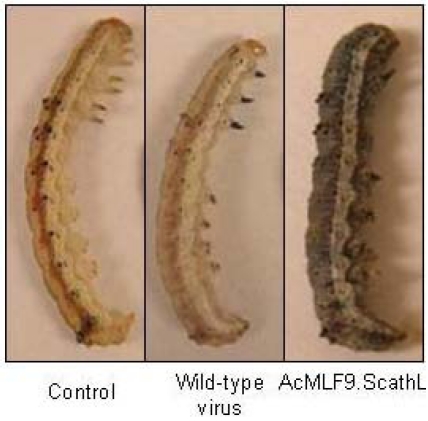
Cuticular melanization resulting from baculovirus expression of ScathL in larvae of *Heliothis virescens*. Control (uninfected) and wild type virus-infected larvae are shown for comparison.

**Figure 3:**
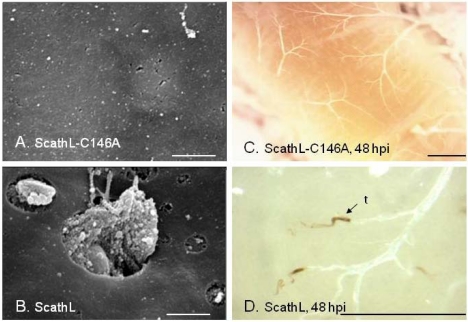
Impact of ScathL on internal tissues of *H. virescens*. A, B, Scanning electron micrographs of the basement membrane overlying the fat body of larvae infected with recombinant baculoviruses expressing the catalytic site mutant ScathL-C146A (A) or wild-type ScathL (B) showing ScathL-mediated perforations. Bars, 2.5 µm. C, D, Light microscopy images of trachea of larvae infected with viruses expressing ScathL-C146A (A) or wild-type ScathL (B) showing melanized tracheal tips (t). Scale bar, 0.5 mm. (Reprinted with permission from Elsevier, [95].)

Fifth instar larvae of *H. virescens* infected with the ScathL-expressing virus AcMLF9.ScathL exhibited a significant degree of cuticlar melanization (Figure 2). Dissection of these larvae also showed patches of melanization over parts of the internal anatomy, as well as fragmentation of some of the tissues [88] (Figure 3). Further experiments with viruses carrying marker genes suggested that ScathL activity did not promote systemic spread of viral infection or alter tissue trophism of the infecting virus [92]. Microscopic examination of tissues in larvae infected with AcMLF9.ScathL showed damage to basement membranes overlying the midgut, fat body, and muscle fibers [93]. Purified ScathL protease was also toxic to insects (specifically, *H. virescens*; the tomato moth, *Lacanobia oleracera*; and the pea aphid, *Acyrthosiphon pisum*) when injected into the hemocoel, and caused melanization in the lepidopteran larvae similar to the melanization seen when ScathL was expressed during baculovirus infection [94,95]. The purified enzyme was capable of causing basement membrane damage *in vivo* and degraded basement membrane proteins *in vitro* [93,94]. Melanization, mortality, and the level of hemolymph cysteine protease activity were closely correlated in lepidopteran larvae infected with AcMLF9.ScathL [95]. 

ScathL may activate the phenoloxidase cascade indirectly by damaging the BM and underlying tissues, or directly by cleaving prophenoloxidase or an upstream serine protease. The total potential phenoloxidase activity (from prophenoloxidase and phenoloxidase combined) was reduced by approximately 50% in wild-type virus infected *H. virescens* larvae and by 75% in AcMLF9.ScathL-infected larvae, while the actual activity from activated phenoloxidase in infected larvae and in non-melanized vs. melanized larvae was unaffected by ScathL expression [95]. Purified ScathL did not activate prophenoloxidase *in vitro* when added to larval hemolymph. Finally, melanization was not observed in aphids or mosquitoes injected with purified ScathL [95]. These results indicate that the toxic effect of ScathL on insects can be explained primarily as a result of the proteolytic damage done to basement membranes and the underlying tissues [93]. The formation of melanin by-products may contribute to its toxicity in lepidopteran larvae.

A homolog of ScathL has been identified from the guts of larvae of the wheat bulb fly, *Delia coarctata* [96]. This protease, DcCathL, is also toxic when injected into the hemocoel of larvae of *L. oleracea*, and seems to be less specific in protein degradation than ScathL. DcCathL selectively degraded serine protease inhibitors (serpins) from the cabbage moth, *Mamestra brassicae*, and *M. brassicae* serpins could block the insecticidal activity of DcCathL when they were co-injected along with DcCathL into the hemocoel of *M. brassicae* larvae. The melanization normally observed with larvae injected with DcCathL was also blocked when the serpins were co-injected, indicating that the serpins could block the proteolytic cascade, presumably initiated by DcCathL-mediated activation of a serine protease in the cascade, that led to prophenoloxidase activation and melanin formation. This result suggests that melanization in larvae injected with DcCathL may be facilitated by the degradation of endogenous serpins by DcCathL. 

A cathepsin B-like protease identified from *Helicoverpa armigera* reduced the survival time of baculovirus-infected *H. armigera* larvae by 12 hr when expressed by a recombinant baculovirus during viral infection [97]. Like the *S. peregrina* cathepsin L-like protease, this cathepsin B played a normal role in the development of *H. armigera* [98,99,100], but had an insecticidal effect when indiscriminately expressed or expressed at levels far above those found under normal physiological conditions. 

The venoms of arthropod predators sometimes contain metalloproteases and gelatinolytic serine proteases that are capable of cleaving basement membrane proteins [101,102,103,104,105,106]. These proteases may also have an insecticidal effect when delivered to the hemocoel of an insect. The reprolysin metalloprotease homolog EpMP3 from the venom of the parasitic wasp *Eulophus pennicornis* [107] caused mortality in *L. oleracea* larvae when purified, recombinant enzyme was injected into the hemocoel. The mortality occurred just prior to or during molting to the next instar, and surviving larvae exhibited a slower rate of growth and development after injection. Soldier-caste nymphs of the social aphid species, *Tuberaphis styraci*, produce a toxic cathepsin B protease in their intestine [108]. When aphid predators threaten the galls produced by reproductive aphids, the soldier nymphs thrust their piercing mouthparts (stylets) into the intruders and orally excrete this cathepsin B, causing paralysis or death of the intruders. Purified recombinant *T. styraci* cathepsin B killed larvae of the wax moth, *G. mellonella*, within 2-4 h when injected into the hemocoel. 

Pathogenic bacteria often express proteases that are toxic to their hosts [109]. *B. thuringiensis* encodes a metalloprotease, InhA, with collagenolytic activity [110]. Genetic studies with *inhA* mutants indicated that it was not required for *B. thuringiensis* virulence [40], but infections of culture filtrates containing InhA caused mortality in waxmoth (*G. mellonella*) larvae.

## 3.  Application of Proteases with Biocontrol Potential

Some of the proteases described above, particularly those that target the PM, act as “stomach poisons” upon ingestion by an insect. However, the toxic effect of proteases that hydrolyze proteins on the cuticle or in the hemocoel generally depends upon an appropriate means to deliver the proteases to their targets. As described above, one popular means to deliver proteases into the insect hemocoel is by means of an insect pathogen, such as entomopathogenic fungi or viruses. However, these proteases can be applied as an insecticide by themselves, without the requirement for an insect pathogen to deliver them to their target sites.

The toxicity of PR1A and other fungal cuticle-degrading enzymes described above occurred when those enzymes were expressed in the hemocoel via a fungal vector. However, significant mortality of an arthropod, the hard tick, *Haemaphysalis longicornis*, could be achieved by topical application of medium from insect cells infected with a recombinant baculovirus that expressed an *H. longicornis* chitinase [111]. Most of the observed mortality could be achieved with medium from which budded virus had been clarified by ultracentrifugation. As it is not expected that the baculovirus used in this study (AcMNPV) would infect and kill ticks, this result suggests that the chitinase alone was toxic to the ticks. Cuticle-degrading proteases such as PR1A also may have toxicity towards insects when applied topically. There is further evidence for this idea as the chitinolytic and proteolytic activities in a *B. bassiana* culture supernatant may cause mortality when the supernatant is sprayed on aphids (*A. gossypii*) [112]. 

In addition, there is substantial evidence that proteins can be transported intact from the guts of insects and related arthropods to the hemocoel [113]. Translocation of protein from the gut to the hemocoel is inefficient, with less than 0.1% to approximately 2% of the protein of interest detected in the hemolymph after ingestion. One promising means for enhancing the degree of protein translocation across the gut is to fuse the protein of interest to a lectin. Fusion proteins consisting of snowdrop lectin, *Galanthus nivalis* agglutinin (GNA) linked to neuropeptide hormones or venom peptide neurotoxins retained the activity of the hormone or neurotoxin portion of the fusion and were transported intact across the gut wall into the hemolymph [114,115,116,117,118]. Insects that were fed diets containing these fusion proteins exhibited reduced growth and mortality. However, it remains to be determined whether this strategy can work with insecticidal proteins such as proteases that are larger than the peptide hormones and neurotoxins employed to date.

## 4. Conclusions

Taken together, proteases represent a group of diverse and relatively unexplored agents for use in insect pest management. A key issue before broad application of such proteases for pest control relates to target specificity. For reduced risk associated with any insect control technology, insect specificity is highly desirable. The use of proteases employed in plant defense against herbivory holds particular promise for future development of insect resistant transgenic plants. Greater understanding of the biology of virulence factors in the genomics and transcriptomics era may facilitate identification of candidate proteases for use in pest management. 
